# A person-centred model of HIV nursing care: translating principles into practice

**DOI:** 10.3389/frhs.2026.1819149

**Published:** 2026-09-01

**Authors:** Michelle Croston, Steve Callaghan, Justine Mellor, Eileen Nixon

**Affiliations:** 1 Central Manchester University Hospitals NHS Foundation Trust, Manchester, United Kingdom; 2 EQE Health, Central Manchester University Hospitals NHS Foundation Trust, Manchester, United Kingdom; 3 Brighton and Sussex Medical School, Brighton, United Kingdom

**Keywords:** HIV, HIV care, HIV nursing, models, person centerd care

## Abstract

This paper presents the development and application of a *Person-Centred Model of HIV Nursing Care* created by the National HIV Nurses Association (NHIVNA). The model responds to the evolving complexities of HIV care and the need to articulate and standardise the nursing contribution within the HIV care continuum. Developed collaboratively with people living with HIV, clinical nurse specialists, and academics, the model provides a structured, person-centred framework to guide high-quality nursing care across diverse clinical contexts.

**Objectives:**

The model aims to:

- Enhance care quality and patient experience through empathy, relational practice, and co-designed care.
- Support nurses' professional development and reduce variation in care.
- Promote workforce sustainability by fostering role clarity, engagement, and shared understanding of the nurse's value in the care pathway.

**Implementation:**

Grounded in McCormack and McCance's person-centred nursing framework, the model adapts these principles to the unique psychosocial dynamics of HIV care, including stigma and self-stigma. Central to the model is empathy and the therapeutic nurse–patient relationship, which enables open communication, psychological safety, and holistic care that addresses physical, emotional, and social dimensions of wellbeing. Implementation of the model of care is supported by a reflective learning workbook designed to integrate the model into clinical practice and continuous professional development. The workbook encourages reflective dialogue, shared learning, and professional growth, aligning with values-based leadership and person-centred culture.

**Conclusion:**

The paper concludes that embedding a person-centred approach within HIV nursing enhances care consistency, improves workforce morale, and builds a sustainable, compassionate culture capable of adapting to the future needs of people living with HIV.

## Background

The care paradigm for HIV has significantly changed over the past 40 years and with these new changes come additional complexities with regards to how care is delivered. Historically nurses have always been central to the HIV response and delivery of care, therefore making nurses ideally placed to respond to the evolving care needs of people living with HIV. The driving force behind the development of the model of care dates to the National HIV Nurses (NHIVNA) conference in 2018. Prior to this time NHIVNA had produced a series of quality and best practice documents (1–6) to support the evolution of nurse led HIV care. However, the identity of HIV nursing was poorly described and there was a need to define the components and minimum standards of HIV nursing care in tandem with national standards and targets. Variation in HIV care and commissioning has been well described in policy and service review documents (7–9). A cohesive model for HIV nursing was further driven by the urgency of a rapidly changing commissioning environment and the need to improve variation in care, qualifications and competencies. In addition, HIV nurses play a central role in getting towards zero new HIV transmissions in the UK by the year 2030. The model builds upon many years of HIV nurses demonstrating their work, academic research (10) and the development of an HIV Community Nursing Model in 2018 (11).

There had historically been a dearth of workforce planning in HIV nursing with sporadic investment in HIV specialist nurses with limited mentoring or support.

The model of care was developed using several different collaborative approaches that involved people with HIV, clinical nurse specialists and academics from across the UK. The development of the model of care acknowledges the importance of the invaluable contribution of other healthcare workers to HIV care, however the scope of model of care's primary focus was to address the care delivery by registered nurses.

The Delphi technique (12) was used in the development of the model to gain consensus on the major segments and the content outline of each segment. The Delphi technique is a structured communication method designed to achieve expert consensus on complex issues through iterative rounds of questions and feedback. Once the segments and content was agreed by the group, experts from the group took a lead in writing each segment with predetermined headings.

Once the document was comprehensive enough for external comments and review, we sent it to patient stakeholder groups (i.e., UKCAB – an organisation who coordinate feedback responses for patient groups). Furthermore, we presented the model at NHIVNA conferences (2022, 2023 and 2024) and BHIVA (2022) to provide opportunities for feedback and further consultation. This provided us a wider set of opinions from a broader group of healthcare professionals and people living with HIV. Comments were collated from written feedback and thematically analysed.

The overall aim of the HIV person-centred model of nursing care is to enable the delivery of high-quality nursing care that meets the evolving needs of people with HIV. The model is designed to be used either as a whole or can be used as separate segments. However, as the model is integrated into clinical practice, we believe that it will enhance care and improve quality of life for people living with HIV. This model was developed to enable HIV nurses to facilitate and improve the delivery of care amongst a moving care landscape. The model was designed to be person-centred by a working group including people living with HIV, to ensure that their voices were heard and reflected within the model (13–15).

As a result of developing the model we believe it will not only support nurses care for people with HIV but also help to reduce any variation in care, improve standards of care and provide a professional framework for NMC revalidation.

## Why is it critical to have a model of nursing?

The debate continues regarding the role of the nurse in HIV care advancing their competencies to cover what was traditionally within the domain of medical roles (10, 16). Recent surveys within the wider nursing profession demonstrate that specialist nursing feels undervalued, which is also linked to this service being under resourced and when specialises nurses leave, a vacuum of knowledge also leaves (10, 16). It is also evident that there is a variety in service profession available for specialist nursing teams.

A model of nursing creates opportunities to answer some of these issues and provides a clear career pathway, enabling nurses to communicate what it is they do to stakeholders, which then allows for improvements in practice, engagement, collaboration, and service delivery. The model of nursing also helps support nurses to develop competencies, links the work nurses do to patient outcomes and quality of care, whilst demonstrating the value nurses bring to the full pathway of care. *Most importantly* the model of care helps People Living with HIV understand how nurses provide care, what they can expect from the nurse and how they can help the care team to co-design and develop care to improve quality to help with their emerging care needs.

## Why a model of nursing care?

Initially, when exploring how we could draw all the existing documents together to articulate the role of the HIV Nurse in the care cascade, we debated on the most appropriate elements that would best capture the nursing contribution to HIV care. In addition, we further debated should we develop a nursing framework or nursing model of care. Eventually, it became evident that we needed to develop a nursing model of care, as when exploring the scholarly literature (17) it was clear that we needed to define what we did as HIV nurse, articulate how we gained our knowledge and skills to practice within a specialist area (18, 19). Whilst being clear about what HIV nursing care should look like. As our overall aim was to define HIV nursing, incorporate values and articulate skills and competencies, we decided that developing a model of nursing care would enable us to do what we need to. See Table 1 for that outlines the differences between a model of nursing care, philosophy and framework.

**Table 1:** Model, philosophy or framework.

Nursing model and description
Nursing Models – are defined to develop and define what nursing is and could be. They describe beliefs, values, and goals of nursing knowledge and skills needed to practise nursing. They offer frameworks to guide practice and education.
Nursing Frameworks – identify the key concepts and describes their relationship to each other and to the phenomena.
Nursing Philosophy – is a statement that outlines a nurse's values, ethics and beliefs, as well as their motivation to be part of the profession. It includes the nurse's perspective regarding their education, practice and patient care.

## Overview of the model of care

The HIV person centred model of nursing care consists of 11 key sections covering the holistic approach of HIV care, these sections include:

1. Testing and adjusting to diagnosis
2. Supporting recovery from HIV-related illness
3. Treatment and interventions
4. Living with HIV and other long-term conditions
5. Complex care and case management
6. Engagement and retention in care
7. Psychological well-being
8. Stigma-related issues
9. Children and young people
10. Sexual and reproductive health
11. Health promotion and prevention

Within each of these sections there is a brief overview of what that section is about, e.g., stigma related issues, the competency level that the nurses are expected to work at, suggested training and a reflective case study.

## The heart of the model of care, person centred care

Historically HIV care has always prided itself of being humanistic and person- centred (13). As HIV care continues to evolve there is a need to define what HIV nursing care should look like to avoid the essence of HIV nursing to be lost or diluted.

When exploring person centered care alongside HIV nursing it was important to identify what was meant by the terminology that was being used. Person -Centered Healthcare (PHC) is a philosophy in which humanistic ideals can be implemented into clinical practice alongside continuing scientific advances (14). PHC includes physical, psychological, emotional, spiritual and the social components of human existence (15). These multiple layers of complexity collectively, not separately add to the biology of the patient and any care given should take into consideration these components.

There are many definitions available as to what person-centred care means within the nursing literature. The most often cited is the person-centred framework by McCormack and McCance (20), which is comprised of four constructs, which are: prerequisites, care processes, person-centred outcomes and the care environment. Whilst McCormack and McCance's framework offers insight into how person-centred care can be developed within nursing, it does not take into consideration the nuances involved within HIV nursing, such as stigma and self-stigma and the impact this can have within the care setting. NHIVNA felt it was important to define person-centred care in order to contextualise the model of care. Our definition is based on the HIV nursing philosophy (17) which articulates the significant role that empathy plays in care delivery and the development of therapeutic relationships. It is through the empathic nurse/patient relationship that concerns can be raised, so that care can focus on the priorities of the patient. Empathic and open communication enables the delivery of person-centred care.

We recognise that stigma and self-stigma are fundamental issues within HIV care and what makes nursing in this context different. However, we feel strongly that stigma should not be placed within the definition, as we want to avoid automatically locating this concept within the individual.

## Implementation into clinical practice

To inform the training needs of the HIV nursing team within the Northwest of England, a working group was established to explore how the model of care could be used to inform teaching and learning as a team. As a result of these discussions a workbook was designed to support HIV nurses professional and personal development using the HIV person centred model of nursing as a framework. Some of the sections within the model covered a variety of different areas of nursing care. The aim of the workbook is to use the structure of the workbook to guide continuous professional development. To gain the most from this workbook learners were encouraged to familiarise themselves with the NHIVNA HIV person centred model of nursing care.

Each section within the workbook was structured to enable the learner to reflect on the theories and concepts discussed within each of the section that are contained within the HIV person centred model of nursing care and consider how this may relate to clinical practice. There is an initial activity that invites the learner to reflect on their current level of competency with regards to that section within the model of care. The main body of the workbook is punctuated with a mixture of reflection points, activity boxes, case studies and resource boxes to aid learning.

The workbook can be used in a variety of different ways. The learner may choose to focus on one section of the model that relate to their own clinical practice and interests, or they may choose to start at the first section of the model and work their way round the circle. Central to the model is person centred care, so we advise that the learner works through the person-centred workbook first and familiarise themselves with what this means in the context of HIV care. Wherever possible the workbook guides the learner to a variety of different type of learning that are linked to the model, however learners are encouraged to add to the workbook and develop/share any resources that they find. To help with this within the workbook we have created some blank reflection templates and additional learning logs to enable the learner to record their learning.

Over a period of 12 months the HIV nursing team across central and south Manchester met to engage in a series of educational sessions that focused on one of the sections from the model of care that linked to the workbook. At the end of the 12-month period the nurses (*n* = 7) were asked to evaluate the educational sessions using the Kirkpatrick Model as an evaluation framework (21–23).

Developed more than 50 years ago, Kirkpatrick's framework for evaluation is one of the most widely used frameworks for evaluating training and learning interventions in healthcare, corporate, and educational settings (24).

Kirkpatrick stressed that an evaluation should go beyond immediate reactions of the attendees, therefore evaluation is carried out on four different levels: 1) reaction 2) learning 3) behaviour and 4) results. However, this is not an evaluation checklist, rather a comprehensive assessment of the four levels to gain a greater insight into the effectiveness of education. Table 2, provides a deeper understanding of what the four levels of measurement mean in the context of evaluation.

**Table 2:** Overview of the Kirkpatrick framework.

Level 1: Reaction – measures participants' immediate feelings, satisfaction, and perceptions of the training. To assess whether learners found the training engaging, relevant, and well delivered. Positive reaction does *not* guarantee learning, but negative reactions often predict poor learning outcomes
Level 2: Learning – measures the degree to which participants acquire the intended knowledge, skills, attitudes, confidence, or competence. To determine whether learning objectives were achieved. Training effectiveness ultimately depends on whether learning occurred
Level 3: Behaviour (Transfer)- measures the extent to which participants apply what they learned when back in their workplace. To determine whether learning has translated into behaviour change. Behaviour change is essential for training to influence real-world practice and patient outcomes.
Level 4: Results- The final outcomes that occur because of the training, often linked to organisational goals. The identifies the real-world impact of the training on service, productivity, or patient care. This level demonstrates the strategic value of training for the organisation.

Reference: Kirkpatrick, (21).

## Results of the evaluation

All seven nurses rated their satisfaction with the training as either 4 or 5 out of 5 indicating a high overall satisfaction with the training. All participants agreed (scores of 4 or 5) that the training was a valuable use of their time. Nurses strongly agreed that learning alongside other HIV nurses was beneficial. All nurses reported feeling their HIV nursing knowledge had increased (scores of 4 or 5). Participants felt more confident addressing holistic needs during consultations. The following were reported as changes in practice because of engaging with the learning. Greater focus on patient-centered care, incorporating case-based learning into teaching sessions, applying research and evidence-based practice, improved consultation structure and motivational interviewing style and finally strengthened collaboration with psychologists and support workers. When asked to explore the impact the training has had on the learners three themes emerged, professional development, impact on patient care and long-term goals.

It is evident from the evaluation that the training was highly effective across all four levels of Kirkpatrick's model (21). There was clear evidence of strong satisfaction and engagement from the learners with the sessions. The educational sessions improved both learners' confidence and knowledge and prompted positive changes within their clinical practice because of attending the sessions. The overall evaluation of the educational sessions has demonstrated evidence of improved person-centred care alongside enhanced professional development.

## Conclusion

As the HIV care landscape continues to evolve, our understanding of what high quality care means in the context of HIV will alter. However, what will remain constant is our underpinning philosophy of ensuring that people living with HIV are at the centre of their care.

The paper demonstrates how a national model of HIV nursing care can be successfully implemented at a local level, using recognised evidence-based frameworks. How empathy and relational practice create psychologically safe spaces for both staff and patients, improving nurse satisfaction and retention.

The development of the workbook and its integration into education and professional development provides actionable strategies for embedding person-centred principles into workforce training and leadership.

## Data Availability

The original contributions presented in the study are included in the article/Supplementary Material, further inquiries can be directed to the corresponding author.
